# An updated review on the link between oral infections and atherosclerotic cardiovascular disease with focus on phenomics

**DOI:** 10.3389/fphys.2022.1101398

**Published:** 2022-12-14

**Authors:** Mariana Bezamat

**Affiliations:** Department of Oral and Craniofacial Sciences, School of Dental Medicine, University of Pittsburgh, Pittsburgh, PA, United States

**Keywords:** cardiovascular disease, oral infections, genetics, phenomics, atheroscelrosis, dental caries, periodontitis (inflammatory)

## Abstract

Atherosclerotic cardiovascular diseases (ACVD) and oral infections such as periodontal disease, dental caries, and apical periodontitis are diseases that affect a great portion of the worldwide population. Both are complex in nature and several studies show that they share etiological factors but a causal relationship between them has not been yet established due to the lack of well-designed clinical trials. Many studies in the recent years show convincing evidence of different mechanisms that might be involved in this association including chronic inflammation and immune response. However, some of these mechanisms are controversial because of confounding factors. It has been suggested that maladaptive inflammatory reactivity, determined in part by single nucleotide polymorphisms (SNPs) in pathway genes, could influence the association between oral infections, and cardiovascular diseases as pleiotropic genes. That is, these conditions could be outcomes of similar inflammatory pathways. Phenomics, the study of the changes in phenotypes or traits considering environmental variables and genetics is efficient in assessing and determining pleiotropic genes. Future research should focus on analyzing a combination of oral inflammatory conditions instead of focusing on one inflammatory phenotype alone and consider pleiotropy as a mechanistic contributor for these associations. Further, prospective observational studies seeking to follow the development of oral disease and subclinical ACVD will foster the understanding of the impact of oral health on general systemic health. Lastly, to demonstrate that oral infections would increase risk for subclinical ACVD development, clinical trials proposing to treat those infections considering genetic background and all other confounders are needed.

## Introduction

Oral infections such as dental caries and periodontal disease are within the most common non-communicable diseases worldwide (World Health Organization, 2015). Dental caries affect the hard tissues of teeth and is the most prevalent chronic disease in both children and adults (Pitts et al., 2021). Periodontal disease is a low-grade chronic inflammatory condition that affects the surrounding structures of teeth (Nwizu et al., 2020) and more than 45% of US adults older than 30 years have periodontal disease (Aarabi et al., 2017). Both have been associated with increased risk of atherosclerotic cardiovascular disease (ACVD) (Kim et al., 2019; Peruzzi et al., 2022). Another type of oral infection that is much less studied is apical periodontitis or periapical lesions. Those lesions appear after bacterial invasion to the dental pulp, contribute to chronic inflammation and periapical bone destruction (Messing et al., 2019).

Atherosclerotic cardiovascular disease (ACVD) is a multifactorial condition characterized by the formation of fibrofatty lesions in the artery wall and is responsible for most myocardial infarctions and strokes (Libby et al., 2019). The artery wall comprises three layers which are the vascular intima, media, and adventitia with atherosclerosis usually starting in the vascular intima and progressing to the medial artery wall (Yu et al., 2014). This deposition of substances including lipids, glycocomponents, and calcium that act to thicken the vessel wall through processes that are mediated by and/or trigger chronic inflammation can lead to atherosclerotic plaque buildup (Peruzzi et al., 2022).

Because of the impact that preventing and treating chronic oral infections could provide in reducing ACVD risk, there are efforts in the recent years to determine the mechanisms of causality between those conditions. However, a causal link has not been yet established (Priyamvara et al., 2020). Environmental factors such as diet, smoking habits, and social determinants of health that contribute to both oral and cardiovascular health must be considered as confounders in the association between oral health and ACVD. Thus, phenomics or the study of phenotypes considering external modifiers and genetics may be an efficient approach for future studies. This review provides an update on the epidemiological association between indices reflective of oral infections and atherosclerosis risk, discusses potential links between those conditions with focus on phenomics, and emphasizes the current knowledge gaps and challenges to inform and guide future studies.

## Studies on the link between oral health and atherosclerosis

### Periodontal disease

To date, periodontal disease is the most studied oral infection in relation to ACVD and most studies focused on the association with clinical outcomes or ACVD events such as myocardial infarction (Park et al., 2019). Although the literature on the link between cardiovascular disease and periodontal disease is abundant, there are few studies focusing on subclinical ACVD and periodontal disease link (Priyamvara et al., 2020). One of the first studies on this subject investigated sex differences in the relationship between periodontal infections and tooth loss and subclinical atherosclerosis (i.e., presence of carotid plaque) (Desvarieux et al., 2004). This study included 1740 participants between the ages of 45 and 75 (mean age 59 ± 8 years) with no history of myocardial infarction or stroke. Their results suggested that long-term periodontitis, defined as probing depth ≥5 mm and attachment loss ≥4 mm, was associated with subclinical atherosclerosis in men but not in women. The authors, however, discuss limitations including the cross-sectional nature of the study and the lack of time sequence of diseases, which did not allow causal inference (Desvarieux et al., 2004).

Contradicting this lack of association between periodontitis and atherosclerosis in women, a smaller study that only included female subjects demonstrated an association between dental plaque, severe gingival inflammation, periodontitis, and carotid intima-media thickness after adjustment for traditional ACVD risk factors (Soder et al., 2007). The study included 46 women with periodontitis and 21 periodontally healthy women and performed an atherosclerotic risk factor analysis and carotid ultrasonography. The results identified periodontitis as a principal-independent predictor of carotid arterial wall thickness. However, it is important to note the potential confounding effects of smoking status (higher in the case group) and educational attainment (higher in the control group) (Soder et al., 2007). Further, a study published in 2022 confirms the association between atherosclerosis, characterized by the mean carotid intima-media thickness and periodontal disease, using a different diagnostic method, the Community Periodontal Index, in both normotensive and hypertensive participants from an urban area in Japan (Kida et al., 2022). Interestingly, another recent powerful population-based cohort study showed that the risk for peripheral artery occlusive disease, a condition with similar atherosclerosis pathogenesis, was increased in patients that had periodontal disease. This same study also found that patients undergoing at least one dental scaling procedure, a treatment for periodontal disease, had lower risk of peripheral artery occlusive disease (Yeh et al., 2022).

Like cardiovascular disease, a variety of indices can be used to ascertain periodontal disease. This lack of uniformity in periodontal disease definitions across epidemiologic studies, such as the ones previously cited, presents a challenge (Nwizu et al., 2020). Further, a new classification of periodontal diseases was recently created (Ndjidda Bakari et al., 2021) and not yet widely used in relationship to ACVD, which creates new opportunities to standardize and assess this association in future studies.

The two proposed mechanisms explaining the link between periodontal disease and ACVD include subgingival pathogens invading endothelial cells directly or an indirect mechanism created by augmented levels of inflammatory cytokines due to periodontitis stimulating a chronic inflammatory response. The first mechanism is supported by the presence of periodontal disease bacteria formed in atherosclerotic lesions in the coronary arteries and the second by the presence of increased levels of IL-1b, IL-6, IL-8, and TNF-a which are cytokines also related to ACVD (Jawien, 2008; Zardawi et al., 2020). However, the precise mechanism involved in this association has not been yet established (Park et al., 2019).

### Dental caries and apical periodontitis

The literature on the association between dental caries and ACVD is less abundant. A 10-year follow-up study conducted with more than 200,000 Korean adults identified an association between dental caries and cardiovascular events (non-fatal stroke and myocardial infarction, heart failure and a composite of cardiovascular mortality) that remained significant after adjustment for demographic and cardiovascular risk factors (Park et al., 2019). However, no assessments of subclinical markers were conducted. There is convincing evidence that shows the presence of *Streptococcus mutans* in cardiovascular specimens, such as heart valves and atheromatous plaques (Soto-Barreras et al., 2013; Fernandes Forte et al., 2022). *Streptococcus mutans* is found in biofilms on tooth surfaces and is a major etiological agent of dental caries (Lemos et al., 2019). Additionally, the only recently published work that assessed subclinical markers of atherosclerosis showed that childhood oral infections, including caries, were associated with adulthood carotid intima media thickness (Pussinen et al., 2019). This last study also highlights the impact that combining oral infections as one phenotype might provide in these types of association studies.

Apical periodontitis or periapical lesion is another type of oral infection, usually caused by caries and characterized by destruction of apical periodontal structures by bacterial invasion into the dental pulp (Messing et al., 2019). With root canal treatment these lesions often regress, however, in some cases asymptomatic bone destruction and chronic inflammation continue developing (Bouillaguet et al., 2018). Both when the apical periodontitis is left untreated or when root canal treatment is unsuccessful, there will be a conflict between individual’s immune response and the microbial pathogens in the dental pulp. This conflict will lead to inflammation and bone resorption, that is, directly proportional to the individual’s immune system capability of fighting the infection (Stashenko et al., 2007)**.** In some extreme cases, tooth extraction is the indicated approach to eliminate these lesions (Karamifar et al., 2020) which highlights the impact of those infections on overall systemic health and potentially on ACVD (Leao et al., 2022).

### Poor oral hygiene and tooth loss

Poor oral hygiene is a major risk factor for the appearance and development of oral infections and inflammation and this increased oral inflammatory burden has been reported to be independently associated with carotid atherosclerotic burden (Leao et al., 2022). Due to poor oral hygiene, pathological bacteria present in the mouth may enter the bloodstream causing bacteremia and bacterial growth over atherosclerotic coronary artery plaques (Priyamvara et al., 2020). A recent cross-sectional study that included 847 older adults (mean age of 70 ± 9 years) from China found that poor oral hygiene was correlated with increased maximal carotid intima-media thickness and atherosclerotic plaque burden in men and women, even those with normal blood glucose levels (Yu et al., 2014). One of the risk factors of both atherosclerotic diseases and oral diseases is type 2 diabetes (Schaefer et al., 2009). Atherosclerotic plaque was identified in 34% of hyperglycemic and 22% of euglycemic participants. Atherosclerotic plaque was also associated with significantly higher number of teeth lost, mean dental plaque index, and loss of gingival attachment as compared with those without atherosclerotic plaque, regardless of glycemic status (Yu et al., 2014).

A study published in 2022, identified an increase in the number of teeth lost in participants with coronary artery disease as compared to participants with no coronary artery disease (Sinha et al., 2022). Additionally, a systematic review and meta-analysis of 44 studies correlating tooth loss to the risk of ACVD and death concluded that lower number of teeth present is a risk factor for ACVD and death and highlighted the importance of good oral hygiene in the prevention of tooth loss (Beukers et al., 2021).

## Oral infections, ACVD, genetics, and phenomics

One of the controversial suggested mechanisms of association between chronic oral infections and ACVD is genetic susceptibility. There is consistent evidence of highly pleiotropic genetic loci associated with ACVD, stroke, type 2 diabetes, Alzheimer’s and also periodontal disease (Sanz et al., 2020). Phenomics, the study of the changes in phenotypes considering environmental variables and genetics is efficient in assessing and determining pleiotropy, a phenomenon in which a single gene or a locus influence multiple traits or diseases (Bezamat et al., 2020). It has been postulated that genetic variants in Fc gamma, human leukocyte antigen (HLA) histocompatibility receptor and TNF-a genes are associated with oral infections (Janket et al., 2015). Oral infections and metabolic inflammation (as seen with atherogenic diet, obesity, and diabetes) share common inflammatory pathways including IL1-b, IL-6, PAI-1, CRP, and TNF-a. Some authors suggest that assessments of associations between oral infection and cardiovascular disease must control for genetics and metabolic inflammation (Janket et al., 2015). Meta-inflammation is a chronic state of low-grade inflammation induced by obesity or long term overnutrition (Li et al., 2018). To determine whether periodontal disease is an independent risk factor for cardiometabolic disease, or a result of underlying meta-inflammation will require continuous evaluation of oral infection, nutrition, gut microbiome, and innate immunity (Janket et al., 2015).

**FIGURE 1:**
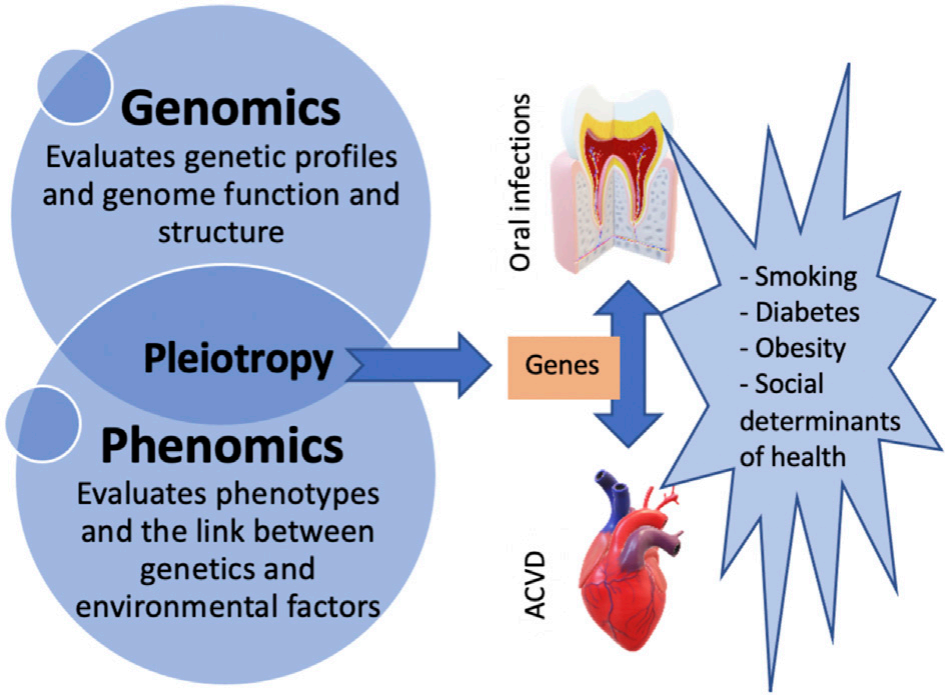
Diagram illustrating the concept of pleiotropy and the association between oral infections, ACVD, and their risk factors.

Loos et al., on the other hand, suggest that aberrant inflammatory reactivity, determined in part by genetic variants in *CDKN2B-ASI (ANRIL), PLG, CAMTA/VAMP3,* and *VAMP8* may modulate the association between periodontitis and cardiovascular diseases as pleotropic genes. In other words, periodontitis is not necessarily causally related to atherosclerosis, but those two conditions are outcomes of similar inflammatory pathways (Loos and Van Dyke, 2020) influenced by similar genes. Identifying pleotropic genes and their effects on ACVD and oral infections would help clarify causal mechanisms between these multifactorial conditions. In addition, determining the genetic variants involved in their pathogenesis will allow future studies to focus on the analysis of specific inflammatory pathways and the adjustment for shared risk factors. Figure 1 illustrates the concepts of phenomics, pleiotropy, and the risk factors contributing to both ACVD and oral infections.

The last assessments of genetic loci associated with both oral infections and ACVD published in 2015 (Schaefer et al., 2015) and in 2017 (Aarabi et al., 2017), only reported 4 genetic loci. A later genome-wide association study identified the *ASIC2* locus as associated with severe gingival inflammation (Zhang et al., 2016). Another study reported *PLG* variants associated with both chronic and aggressive periodontitis (Munz et al., 2017). Both genes have been suggested to also play a role in atherosclerosis (Wang et al., 2016; Bonomi et al., 2020).

## Discussion

Improved oral hygiene has been shown to improve blood biomarkers of systemic inflammation including hsCRP, MCP-1, and IL-6 potentially also influencing atherogenesis (Gianos et al., 2021). Subclinical markers of atherosclerosis such as carotid intima media thickness and periodontitis (Ahn et al., 2016) and coronary artery calcium and tooth loss (Donders et al., 2020) have been recently suggested as suitable markers for association studies between these diseases. However, different variables and confounders must be considered in the analyses of these associations and standardized methods to ascertain phenotypes must be adopted. The new periodontal disease classification opens up new avenues for future studies. In addition, combining oral inflammatory phenotypes proves appropriate to determine and assess a more homogeneous overall oral inflammatory burden with less confounders.

The American Heart Association measures cardiovascular health with a checklist of 8 risk factors which are known as the “Life’s Essential 8” including diet, physical activity, nicotine exposure, body mass index, blood pressure, lipids, glucose, and the recently added 8th risk factor: sleep duration. As suggested before, oral health traits such as periodontal or gum disease, caries, or significant loss of teeth could potentially serve as efficient markers of ACVD risk allowing dentists to play a greater role in the prevention and improvement of such disorders (Tremblay et al., 2011). If research could determine a definite association between oral health and ACVD and oral health becomes one of the “life’s essential” risk factors, a greater awareness, importance, and attention would be given to the prevention and early treatment of oral infections potentially impacting cardiovascular outcomes.

More studies are needed to determine the genetic link between oral health phenotypes and ACVD as well as establish all shared genetic risk loci involved in these inflammatory conditions. Phenome-wide approaches are efficient for studies aiming to determine pleotropic effects or discard shared genetic associations. This approach is helpful in shading light on possible mechanistic pathways considering environmental factors that might also be involved.

In summary, primary prevention of ACVD is possible and future studies should focus on assessing subclinical markers of ACVD and oral health impact on their development. Clinical trials demonstrating that treating oral infections reduce ACVD events and risk are suggested although costly and ethically complicated to be designed. A study design proposing to assess subclinical markers of ACVD and how they can improve in response to oral infections control and treatment may be more feasible and impactful because if proven effective, it would possibly allow for primary prevention of ACVD.
